# Complications in defibrillator surgery in patients with implanted ventricular assist device

**DOI:** 10.1186/1749-8090-10-S1-A214

**Published:** 2015-12-16

**Authors:** Thomas Schroeter, Sven Lehmann, Mahmoud Sleiman Wehbe, Maximilian Vondran, Philipp Kiefer, Martin Misfeld, Friedrich Wilhelm Mohr, Jens Garbade, Anna Meyer

**Affiliations:** 1 Heart Center Leipzig, University of Leipzig, Department of Cardiac Surgery, Struempellstrasse, 39 04289, Leipzig, Germany

## Background/Introduction

The implantation and exchange of pacemakers and defibrillators (ICD) is meanwhile a fully developed method with very low complication rates. However, patients with the need for a permanent anticoagulation with warfarin have a significantly increased risk for bleeding in the intraoperative and postoperative course

## Aims/Objectives

The aim of this study was to evaluate bleeding complications associated with implantation or replacement of implantable cardioverter-defibrillators (ICD) in patients with a ventricular assisted device (VAD) in whom continuous anticoagulation is required.

## Method

A total of 37 patients with a VAD in whom an ICD has been implanted, explanted or replaced, were retrospectively analyzed.

## Results

All ICD related procedures were successfully performed. There were no procedure-related deaths. Perioperative anticoagulation was achieved with intravenous heparin, which was discontinued four hours prior to surgery and restarted four hours after surgery. The average international normalized ratio ± standard deviation (INR ± SD) was 1.5 ± 0.3, and the mean partial thromboplastin time (PTT) ± SD was 67.1 ± 23.1 seconds. Platelet inhibition in 23 patients (62.2%) was achieved with aspirin, and in 14 patients (37.8%) with clopidogrel up to the time of surgery. The thirty-seven ICD interventions included ten primary implants (27.0%), twenty-three replacements (62.2%) and four explants (10.8%). In a follow-up period of at least three months, a total of nine complications (24.3%) were attributable to ICD intervention. These included two conservatively treated hematomas and six hematomas that required surgical evacuation. There was no difference in bleeding frequency comparing subcutaneous and subpectoral approach. There were no ICD lead damages or thromboembolic events identified in the follow-up period.

## Discussion/Conclusion

With regards to anticoagulation in VAD patients post-ICD intervention, the incidence of hematoma was significantly higher than in non-VAD patients. On the sixth postoperative day (5.7 ± 4.9), the hematoma followed. This delayed incidence of bleeding suggests that re-initiation of warfarin post-ICD intervention in supplement with intravenous heparin represents a vulnerable period in which the ICD-pocket wound tends to bleed. An increased incidence of thromboembolic events was not observed.

